# Investigating the Use of Dry Matter Intake and Energy Balance Prepartum as Predictors of Digestive Disorders Postpartum

**DOI:** 10.3389/fvets.2021.645252

**Published:** 2021-09-16

**Authors:** Johanny Pérez-Báez, Carlos A. Risco, Ricardo C. Chebel, Gabriel C. Gomes, Leandro F. Greco, Sha Tao, Izabella M. Toledo, Bruno C. do Amaral, Marcos G. Zenobi, Natalia Martinez, Geoffrey E. Dahl, Jorge A. Hernández, Jessica G. Prim, José Eduardo P. Santos, Klibs N. Galvão

**Affiliations:** ^1^Escuela de Medicina Veterinaria, Facultad de Ciencias Agronómicas y Veterinarias, Universidad Autónoma de Santo Domingo, Santo Domingo, Dominican Republic; ^2^Department of Large Animal Clinical Sciences, D. H. Barron Reproductive and Perinatal Biology Research Program, University of Florida, Gainesville, FL, United States; ^3^Department of Animal Sciences, University of Florida, Gainesville, FL, United States; ^4^D. H. Barron Reproductive and Perinatal Biology Research Program, University of Florida, Gainesville, FL, United States

**Keywords:** dry matter intake, energy balance, digestive disorders, predictive models, dairy cows

## Abstract

One objective was to evaluate the association of dry matter intake as a percentage of body weight (DMI%BW) and energy balance (EB) prepartum and postpartum, and energy-corrected milk (ECM) postpatum with digestive disorders postpartum. For this, ANOVA was used, and DMI%BW, EB, and ECM were the outcome variables, and left displaced abomasum (LDA), indigestion, and other digestive disorders (ODDZ) were the explanatory variables. The main objective was to evaluate prepartum DMI%BW and EB as predictors of digestive disorders. For this, logistic regression was used, and LDA, indigestion, and ODDZ were the outcome variables and DMI%BW and EB were the explanatory variables. Data from 689 cows from 11 experiments were compiled. Left displaced abomasum was not associated with prepartum DMI%BW or EB. Postpartum data were normalized to the day of the event (day 0). Cows that developed LDA had lesser postpartum DMI%BW on days −24, −23, −12, −7 to 0 and from days 1 to 8, 10 to 12, and 14 and 16, lesser postpartum EB from days −7 to −5, −3 to 0, and 12, and lesser postpartum energy-corrected milk on days −19, −2, −1, 0, 7, 9, 10, 15, and 17 relative to diagnosis than cows without LDA. Cows that developed indigestion had lesser prepartum DMI%BW and EB than cows without indigestion, and lesser postpartum DMI%BW on days −24, −1, 0, 1, and 2, and greater DMI%BW on day 26, lesser ECM on days −24, −2, −1, 0, 1, and 2 relative to diagnosis. Postpartum EB was not associated with indigestion postpartum. Cows that developed ODDZ had lesser prepartum DMI%BW on day −8 and from days −5 to −2, lesser prepartum EB on day −8 and from days −5 to −2, and lesser postpartum DMI%BW than cows without ODDZ. Each 0.1 percentage point decrease in the average DMI%BW and each Mcal decrease in the average EB in the last 3 days prepartum increased the odds of having indigestion by 9% each. Cutoffs for DMI%BW and EB during the last 3 days prepartum to predict indigestion were established and were ≤1.3%/day and ≤0.68 Mcal/day, respectively. In summary, measures of prepartum DMI%BW and EB were associated with indigestion and ODDZ postpartum and were predictors of indigestion postpartum, although the effect sizes were small.

## Introduction

The transition period in dairy cows is characterized by changes in the dry matter intake (DMI). Dairy cows start to decrease their DMI during the last 10 days of gestation, with a pronounced decrease during the last 3–4 days prepartum (1–3). There is an increase in DMI during the first weeks postpartum, although it does not meet the energy requirements for maintenance and milk production; therefore, dairy cows experience a negative energy balance that leads to an increase in non-esterified fatty acids and beta-hydroxybutyrate in blood (4–6).

The postpartum period is also characterized by an increase in the incidence of diseases and disorders that affect the welfare, production, reproduction, and longevity of cows in the herd. Previous research has shown that digestive disorders (i.e., left displaced abomasum (LDA), indigestion, diarrhea, rumen stasis, or bloat) are associated with delayed resumption of ovarian cyclicity (7), decreased fertility (8), and decreased milk yield (9), thus causing economic losses to the herd.

**Figure 1:**
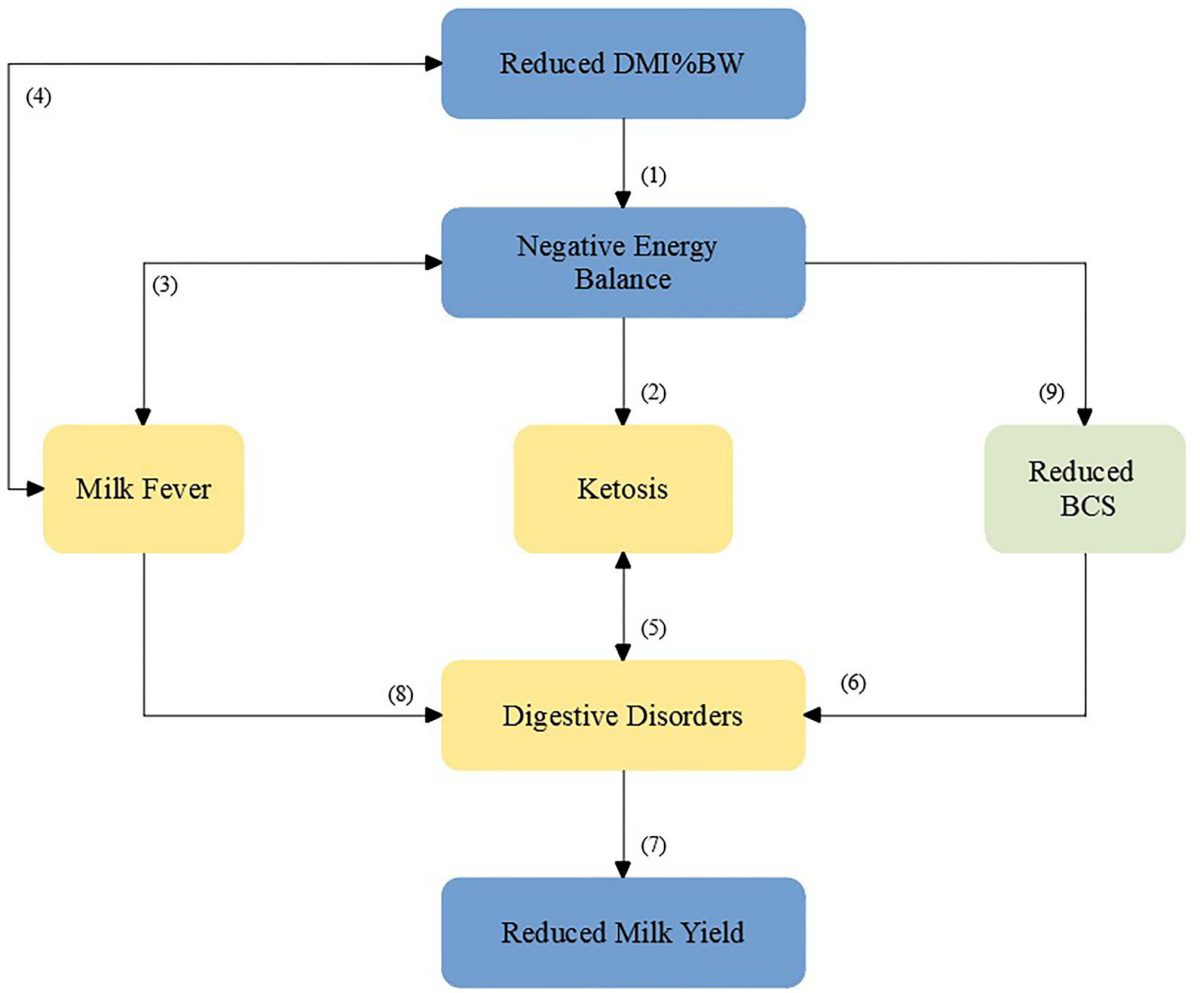
Causal diagram showing the relationship of dry matter as percentage of body weight (DMI%BW) and energy balance with digestive disorders and its consequences. Based on the work of the following: (1) Hammon et al. (19); (2) Ospina et al. (17); (3) Martinez et al. (20); (4) Martinez et al. (21); (5) Raboisson et al. (22); (6) Chebel et al. (18); (7) Raizman et al. (23); (8) Madison and Troutt (24); (9) Hayirli et al. (1).

Several studies have investigated how dry matter and energy restriction prepartum influence dry matter intake and energy balance postpartum, with mixed results. Some studies showed that cows that were feed restricted during the dry period had increased DMI and energy intake in the first 3 weeks postpartum (10), some studies showed mixed results (11–13), and some studies did not show any significant improvements for the feed-restricted groups (14, 15). The literature investigating the association between DMI or energy balance (EB) prepartum and digestive disorders postpartum is more limited. One study showed that cows that were feed restricted during the dry period had lesser incidence of LDA postpartum than cows that were fed *ad libitum* (16). Furthermore, Ospina et al. (17) observed an association between high blood non-esterified fatty acids (NEFA) prepartum and LDA postpartum. In addition, indigestion (described later) has been associated with loss of BCS during the dry period (18); therefore, it is likely that cows with digestive disorders postpartum would have experienced a more severe drop in DMI and EB prepartum. Therefore, the hypothesis of this study is that cows with digestive disorders postpartum would have experienced a greater reduction in DMI as percentage of body weight (DMI%BW) or EB prepartum and would have a lesser DMI%BW or EB postpartum than cows without digestive disorders (Figure 1). One objective was to evaluate the association of DMI%BW and EB prepartum and postpartum, and energy-corrected milk (ECM) postpatum with digestive disorders [indigestion, LDA, and other digestive disorders (ODDZ; described later)]. The main objective was to evaluate the use of prepartum DMI%BW and EB as predictors of digestive disorders postpartum.

## Materials and Methods

### Experimental Design, Housing, and Sample Size

A retrospective longitudinal study was performed using the data from a total of 689 cows (236 primigravid and 453 multigravid) from 11 different experiments conducted at the University of Florida dairy unit, located in the city of Hague, Florida. This was a convenience sample; therefore, no *a priori* sample size calculation was performed. For continuous variables, 122 cows affected with indigestion (described later; Table 1) would provide sufficient power to detect statistical differences with an effect size of 0.25 (e.g., difference in DMI%BW of 0.25 and SD of 1), alpha of 0.05, and beta of 0.2. With a sample size of 26 to 27 cows (LDA and ODDZ; Table 1), only differences with an effect size of 0.5 would be found statistically significant. Individual experiments were approved by the University of Florida Animal Research Committee.

**Table 1:** Frequency table of digestive disorders during the first 28 days postpartum in 689 cows.

**Disorder**	**Frequency**	**Percentage (%)**	**DPP (range)**
Digestive disorders	175	25.4	10 (0–28)
Left displaced abomasum	26	3.7	11 (2–27)
Indigestion	122	17.1	11 (1–28)
Other digestive disorders	27	4.5	
Sand impaction	3	0.4	4 (3–4)
Cecal dilatation	1	0.1	21 (–)
Diarrhea	18	3.2	16 (1–13)
Bloat	3	0.3	6 (4–7)
Constipation	2	0.2	2 (–)

*DPP, average of days postpartum when the disorder was diagnosed*.

The University of Florida dairy unit milked ~500 Holstein cows twice daily with a rolling herd average of ~10,500 kg/cow. The freestall beds and walking alleys were cleaned twice daily. Clean and dry sand was added on the top of the freestall beds twice weekly. Fans with misters and sprinklers over the feed line were present in the barns and activated when environmental temperatures rose above 18°C. Primiparous and multiparous cows were housed separately. Cows were vaccinated and treated for common diseases or disorders according to the standard operating procedures developed with participation of the veterinarians from the University of Florida, College of Veterinary Medicine, Food Animal Reproduction, and Medicine Service.

The experiments were conducted from 2007 to 2017. Six of the 11 experiments were conducted during the hot months (June to October) to evaluate the effect of evaporative cooling during the dry period on production measures (25–30). For these experiments, cows were provided with shade only or with shade plus evaporative cooling with fans and sprinklers. The average environmental temperature during the 3 weeks before calving for these experiments was 26.9°C ± 2.0°C and a temperature humidity index (THI) of 77.7 ± 2.8. Herein, we maintained the categorization of heat stress abatement applied in these six previous experiments resulting in cows categorized as hot with evaporative cooling (*n* = 108) or hot without evaporative cooling (*n* = 106). In the remaining studies, prepartum cows were enrolled from December to May with an average environmental temperature of 17.6°C ± 3.4°C, and a THI of 63.8 ± 8.9 (31–35), and cows were provided evaporative cooling with fans and sprinklers when temperatures rose above 20°C. Fans and sprinklers were turned on and off automatically based on thermostat reading. Fans stayed on while environmental temperature exceeded 20°C, but sprinklers were on cycles of 1 min on and 3 min off. Cows enrolled in the experiments from December to May could still be exposed to heat stress. As far as we know, a heat stress THI cutoff for the dry period has not been established; therefore, we chose a prepartum cutoff of THI ≥70 as the midpoint between the traditional (72) and revised (68) THI cutoffs for lactating dairy cows (36, 37). Hence, we categorized cows as hot with evaporative cooling when the average THI during the last 3 weeks prepartum was ≥70 (*n* = 126 cows) and cool when the average THI for the last 3 weeks prepartum was <70 (*n* = 349). Hence, to account for any conditional effect of heat abatement, the variable heat stress abatement was created: cool, hot without evaporative cooling, and hot with evaporative cooling. The following formula was used to calculate the THI, according to (38):

THI = 0.8° ambient temperature + [(relative humidity/100) × (ambient temperature – 14.3)] + 46.4

The meteorological data obtained from The Weather Underground, Inc. (39) for the city of Hague, Florida was used to calculate THI.

### Measurement of Dry Matter Intake

Cows had their daily DMI recorded using a system with individual feeding gates (Calan Gates, American Calan Inc., Northwood, NH). For this study, we used DMI collected from days −21 to −1 prepartum and from days 1 to 28 postpartum. Dry matter intake on the day of calving (day 0) was not included because of inconsistent DMI measurements due to parturition itself and due to pen moves from the prepartum pen to the postpartum pen. Chemical composition of diets of each experiment included in this study is on Supplementary Table 1.

### Milk Yield and Energy-Corrected Milk

Cows were milked twice a day, and milk production was recorded automatically using milk meters (AfiFlo; S.A.E. Afikim). Data for milk components such as concentrations of fat, true protein, and lactose were available either daily (*n* = 356 cows) or weekly (*n* = 120 cows). For cows sampled weekly, daily measurements were estimated by interpolation. Milk fat percentage decreases linearly from weeks 1 to 4 of lactation (40); therefore, interpolation would be an acceptable method for estimating daily fat percentage. As an example, when fat percentage was available for day 7 (Fat % = 3.12) and day 14 (Fat % = 3.55) postpartum, fat percentage on each subsequent day from days 7 to 14 was calculated using the formula: Fat percentage (Fat %) subsequent day = [(Fat % day 14 – Fat % day 7)/7] + Fat % previous day. For day 8, Fat % day 8 = [(3.55 – 3.12)/7] + Fat % day 7 = 0.06 + 3.12 = 3.18%. The ECM was calculated as follows, derived from the Nutrient Requirements of Dairy Cattle [NRC, (41)]:

ECM = [(0.3246 × kg of milk) + (12.86 × kg of fat) + (7.04 × kg of protein)].

### Energy Balance

The EB was calculated using NRC (2001) equations for energy requirements as follows:

For prepartum EB:

EB prepartum = Net energy of lactation (NEL) intake – (NEL pregnancy + NEL maintenance)

For postpartum EB:

EB postpartum = NEL intake – (NEL maintenance + NEL milk)

where NEL intake, NEL maintenance, NEL pregnancy, and NEL milk were calculated as follows:

NE intake = DMI × NEL of the diet

NEL maintenance = (BW 0.75 ×0.08)

NEL pregnancy = [(0.00318 × day of gestation – 0.0352) × (calf BW/45)]/0.218.

NEL milk = (9.35 × milk yield × fat percentage/100) + (5.35 × milk yield × protein percentage/100) + (3.95 × milk yield × lactose percentage/100).

### Health Disorders

Detailed paper and electronic health records were recorded for each cow. Each cow underwent scheduled complete physical examinations by a trained herdsman or by a veterinarian from the College of Veterinary Medicine Food Animal Reproduction and Medicine Service (FARMS) at the University of Florida on d 4, 7, and 12 postpartum. Furthermore, the attitude of cows was monitored daily pre- and postpartum, and milk yield was monitored postpartum. Any cow showing signs of depression, inappetence, lethargy, altered stride, inflammation of the mammary gland, or a drop >10% in milk yield underwent a physical examination by a trained herdsman or by a FARMS veterinarian. The veterinarians from FARMS performed physical examinations and provided supervision and training of herd personnel performing clinical diagnosis and treatment of postpartum cows at least once a week. Additionally, FARMS veterinarians were called to assist or confirm clinical diagnosis or treatment of postpartum cows throughout the weekdays and weekends. Only health events occurring during the first 28 days in milk were used in this study. We first retrieved the electronic health records, and then confirmed the information using the paper health records. Cows with mismatched information or with a disease diagnosis prepartum were excluded from the study. The health disorders recorded were ketosis, digestive disorders, calving disorders (dystocia, twins, stillbirths), retained placenta, metritis, and mastitis. Digestive disorders included LDA, indigestion, and ODDZ such as sand impaction, cecal dilatation, diarrhea, bloat, and constipation. Left displaced abomasum was diagnosed by a characteristic ping over the 9th to 13th ribs on the left side and was confirmed during surgery. None of the cows followed were diagnosed with right displaced abomasum. Indigestion was diagnosed in cows with undigested feces (presence of large amount of undigested fiber and grain in feces), scant pasty malodorous feces, rumen stasis (<1 rumen contraction/min), or a combination of two or more of these signs. Sand impaction was diagnosed during surgery in some cows that were suspected to have LDA. Cecal dilatation was diagnosed *via* rectal palpation and was characterized by a caudal displacement of the dilated cecum as previously described (42). Cows with cecal dilatation usually present with abnormal demeanor, decreased ruminal motility, scant feces, and colic. Cecal dilatation may evolve into volvulus and lead to death (42). Cecal dilatation was corrected surgically. Diarrhea was diagnosed in cows with watery feces that would sift through bedding (43). Bloat was diagnosed in cows with gas-distended rumen. Constipation was diagnosed in cows with very dry feces. The diagnosis of some of the clinical signs of digestive disorders such as undigested feces, scant pasty malodorous feces, and constipation can be subjective; therefore, a potential for misdiagnosis exists. Detailed information about calving and uterine disorders and ketosis and mastitis are presented in Pérez-Báez et al. (2, 3). Cows suffering from ketosis, digestive disorders, metritis, or mastitis were treated according to the farm standard operating procedure^1^.

### Statistical Analysis

To evaluate the association of prepartum and postpartum DMI%BW and EB with digestive disorders, we analyzed the data using ANOVA for repeated measures using the MIXED procedure of SAS version 9.4 (SAS Institute Inc., Cary, NC). The data were divided into two periods, prepartum and postpartum. For prepartum, the outcome variables were prepartum DMI%BW or EB, and the explanatory variable was one of the three digestive disorders (LDA, indigestion, ODDZ), and they were modeled separately; cows that developed LDA were compared with cows that did not develop LDA. Cows that did not develop LDA could have developed any other disorder. Cows that developed indigestion were compared with cows that did not develop indigestion. Cows that did not develop indigestion could have developed any other disorder. Cows that developed ODDZ were compared with cows that did not develop ODDZ. Cows that did not develop ODDZ could have developed any other disorder. Other studies have used healthy cows as the comparison group (44). However, this would introduce selection bias; therefore, this could artificially increase the differences in the measures of DMI between the groups and inflate the estimates in a prediction model. The models included the fixed effects of digestive disorder of interest (yes vs. no), parity (primigravid vs. multigravid), BCS in the last week prepartum (<3.75 vs. ≥3.75), day relative to calving (prepartum: days −21 to −1), heat stress abatement (cool vs. hot without evaporative cooling vs. hot with evaporative cooling), and the interaction between the digestive disorder of interest and day relative to calving. Cow was nested within experiment as a random effect. First-order autoregressive, compound symmetry, and unstructured covariance structures were tested, and the first-order autoregressive was selected because it resulted in the smallest Aikaike's information criterion.

As an example, the initial model to evaluate the association between prepartum DMI%BW and LDA was:

DMI%BW prepartum = LDA + day + heat stress abatement + BCS + parity + LDA × day + LDA × season + LDA × BCS + LDA × parity + cow (experiment).

The disorder of interest was forced into the model, but other variables were removed from the model by stepwise backward elimination according to Wald statistics criterion when *p* > 0.05. When an interaction was detected, the mean separation was assessed using the SLICE option in the MIXED procedure, and multiple comparisons were performed using the Tukey-Kramer adjustment method in SAS. All models were tested for multicollinearity using the GLM procedure of SAS, and all variables had a variance inflation factor of <5, therefore, indicating no multicollinearity. It is important to note that these analyses were used to test for associations and by no means can be used to infer causation.

To evaluate the use of prepartum DMI%BW and EB as predictors of digestive disorders, each disorder was considered the dependent variable and DMI%BW and EB as independent variables. These data were analyzed by logistic regression with the GLIMMIX procedure of SAS. The objective was to assess if measures of prepartum DMI%BW or EB were associated with the odds of digestive disorders. In this case, each disease or disorder was the dependent variable and the measures of prepartum DMI%BW or EB were assessed separately in different models as independent variables. For this purpose, the variable average DMI%BW or EB in the last 14, 7, and 3 days prepartum and reduction from days −8 to −1 and −4 to −1 were created. Univariable and multivariable models were performed. The univariable models included cow nested within experiment as a random variable. Measures of DMI%BW or EB with *p* < 0.20 were selected for inclusion in the multivariable logistic regression models. Multivariable models also included parity (primigravid vs. multigravid), prepartum BCS [<3.75 vs. ≥3.75 (45)], and heat stress abatement (cool vs. hot without evaporative cooling vs. hot with evaporative cooling), and cow nested within experiment as a random effect. Two-way interaction terms of measures of DMI%BW and EB with *p* ≤ 0.05 and other covariates were tested. A stepwise backward elimination was performed and explanatory variables with *p* > 0.05 according to the Wald statistics criterion were removed from the model.

When a measure of DMI%BW or EB prepartum had *p* ≤ 0.05, we assessed their contribution to the predictive ability of the logistic regression model containing other covariates by comparing the area under the curve (AUC) of a receiver operating characteristic curve (ROC) of the model with and without the measures of DMI%BW or EB using the ROCCONTRAST statement of the LOGISTIC procedure of SAS as previously reported (46). The AUC ≤0.50 was considered non-informative, AUC between 0.50 and 0.70 was considered with low accuracy, AUC between 0.70 and 0.90 was considered accurate, and AUC between 0.9 and 1.0 was considered highly accurate (47). Finally, we determined cutoff values for measures of DMI%BW and EB prepartum with *p* ≤ 0.05 for predicting digestive disorders postpartum using ROC, and the cutoff with the greatest Youden's J statistic which combines the values for sensitivity and specificity was chosen. The sensitivity, specificity, and overall accuracy of applying the cutoff to predict digestive disorders were calculated. Statistical significance was considered when *p* ≤ 0.05.

For postpartum, data were collected for the first 28 days postpartum and were organized to evaluate the association of DMI%BW, EB, and ECM relative to the day of diagnosis (i.e., days −2, −1, 0 (day of diagnosis), 1, and 2). Therefore, cows diagnosed on day 3 postpartum had 2 days of data before diagnosis and 25 days of data after diagnosis, whereas cows diagnosed on day 26 postpartum had 2 days of data after diagnosis and 25 days of data before diagnosis. Cows that had at least one digestive disorder were matched with cows that did not have the digestive disorder being analyzed but they could have any other disorder. Cows were matched on study number, heat stress abatement treatment, and parity group. Only one cow without a disorder was selected for each cow with a disorder; therefore, if more than one cow fit the matching criteria, an online random selector program (i.e., https://miniwebtool.com/random-picker/) was used to select the matching cow. In this analysis, the outcome variables were postpartum DMI%BW, EB, and ECM, and the explanatory variable was one of the three digestive disorders (LDA, indigestion, ODDZ), and they were modeled separately. The models included the fixed effects of digestive disorder of interest (yes vs. no), day relative to diagnosis, and the interaction between the digestive disorder of interest and day relative to diagnosis. Similar to prepartum data, cow was nested within experiment as a random effect. First-order autoregressive, compound symmetry, and unstructured covariance structures were tested, and the first-order autoregressive was selected because it resulted in the smallest Aikaike's information criterion.

As an example, the initial model to evaluate the association between postpartum DMI%BW and LDA was:

DMI%BW postpartum = LDA + day of diagnosis + LDA × day of diagnosis + cow (experiment).

## Results

The frequencies of each digestive disorders diagnosed during the first 28 days postpartum are shown in Table 1.

### Association of Prepartum DMI%BW and EB With LDA

**Figure 2:**
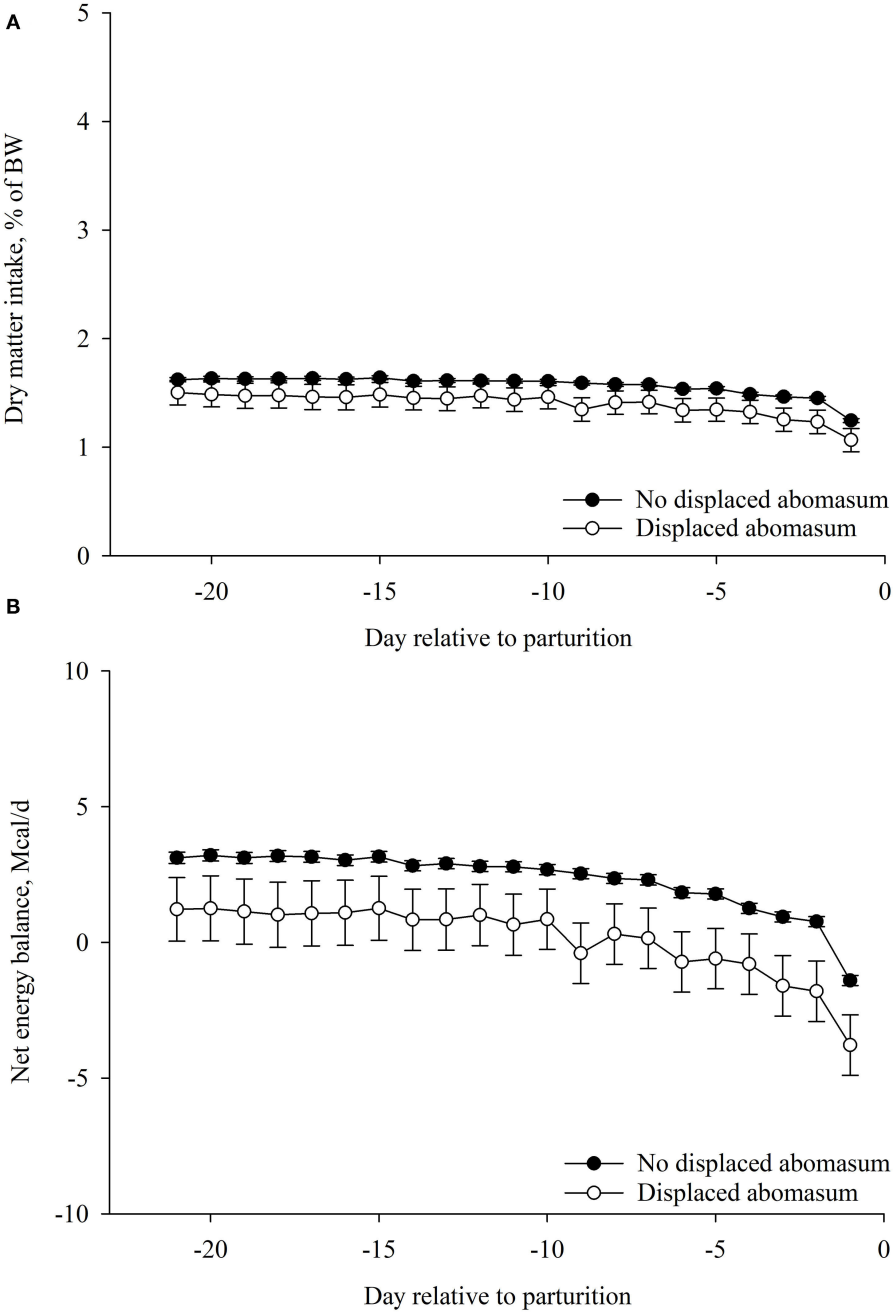
Association of left displaced abomasum postpartum (*n* = 26) with **(A)** dry matter intake (DMI, %BW) and **(B)** net energy balance (EB, Mcal/day) during the prepartum period (from −21 to −1 day relative to parturition). Values are least square means ± SEM. Prepartum DMI (%BW): left displaced abomasum, *p* = 0.15; day relative to parturition, *p* < 0.01; and the interaction between left displaced abomasum and day, *p* = 0.99. Prepartum net energy balance: left displaced abomasum, *p* = 0.03; day relative to parturition, *p* < 0.01; and the interaction between left displaced abomasum and day, *p* = 0.99. *p ≤ 0.05.

Prepartum DMI%BW was not associated with LDA postpartum (*p* < 0.15; Table 2; Figure 2A). Cows that had LDA had lesser prepartum EB (*p* = 0.03) compared with cows that did not have LDA (Table 2; Figure 2B).

**Table 2:** Association of pre- (−21 to −1 day) and postpartum (1 to 28 days) dry matter intake as percentage of body weight (DMI%BW), energy balance (EB), and energy-corrected milk (ECM) with left displaced abomasum (LDA) postpartum according to multivariable analysis.

	**Prepartum**		***p*** **-Value**			**Postpartum**		***p*** **-Value**		
	**LDA**	**No LDA**	**LDA**	**Day**	**LDA × day**	**LDA**	**No LDA**	**LDA**	**Day**	**LDA × day**
DMI%BW	1.47 ± 0.09	1.59 ± 0.02	0.15	<0.01	0.99	2.00 ± 0.22	2.53 ± 0.22	0.11	<0.01	<0.01
EB (Mcal/day)	0.4 ± 0.9	2.4 ± 0.2	0.03	<0.01	0.99	−11.9 ± 1.7	−8.6 ± 1.7	0.28	<0.01	<0.01
ECM (kg/day)	–	–	–	–	–	23.9 ± 4.4	34.3 ± 4.4	0.17	<0.01	<0.01

*Day, day relative to parturition; LDA × day, interaction between left displaced abomasum and day*.

### Prepartum DMI%BW and EB as Predictors of LDA

The average DMI%BW and EB during the last 3 days prepartum were not explanatory variables for LDA (Table 3). Of the variables evaluated, parity was the only predictor of LDA postpartum. Multigravid cows had 9.3 times the odds of developing DA postpartum compared with primigravid cows (OR, 9.3; CI, 2.1–41.7; *p* < 0.01).

**Table 3:** Effect of the average DMI as a percentage of body weight (DMI%BW) and the average energy balance (EB) in the last 3 days prepartum on postpartum left displaced abomasum (LDA), indigestion, and other digestive disorders in the first 28 days postpartum.

	**DMI%BW**			**EB (Mcal/day)**		
**Disorder**	**OR^a^**	**95% CI**	***p*-Value**	**OR^b^**	**95% CI**	***p*-Value**
LDA	1.03	0.95–1.13	0.45	1.03	0.94–1.12	0.58
Indigestion	1.09	1.04–1.15	<0.01	1.09	1.04–1.14	<0.01
ODDZ	1.08	0.99–1.18	0.07	1.08	0.99–1.16	0.08

*ODDZ, other digestive disorders include sand impaction, cecal dilation, bloat, diarrhea, and constipation*.

a *The odds ratio (OR) represents a 0.1 percentage point decrease in the average DMI%BW in the last 3 days prepartum, when the average DMI%BW ranged from 0.27% to 2.90%, with an interquartile range from 1.03 to 1.70%*.

b *The OR represents a unit decrease in the average EB in the last 3 days prepartum, when the average EB ranged from −16.72 to 25.12 Mcal/day, with an interquartile range from −0.82 to 5.47 Mcal/day*.

### Association of Postpartum DMI%BW, EB, and ECM With LDA

**Figure 3:**
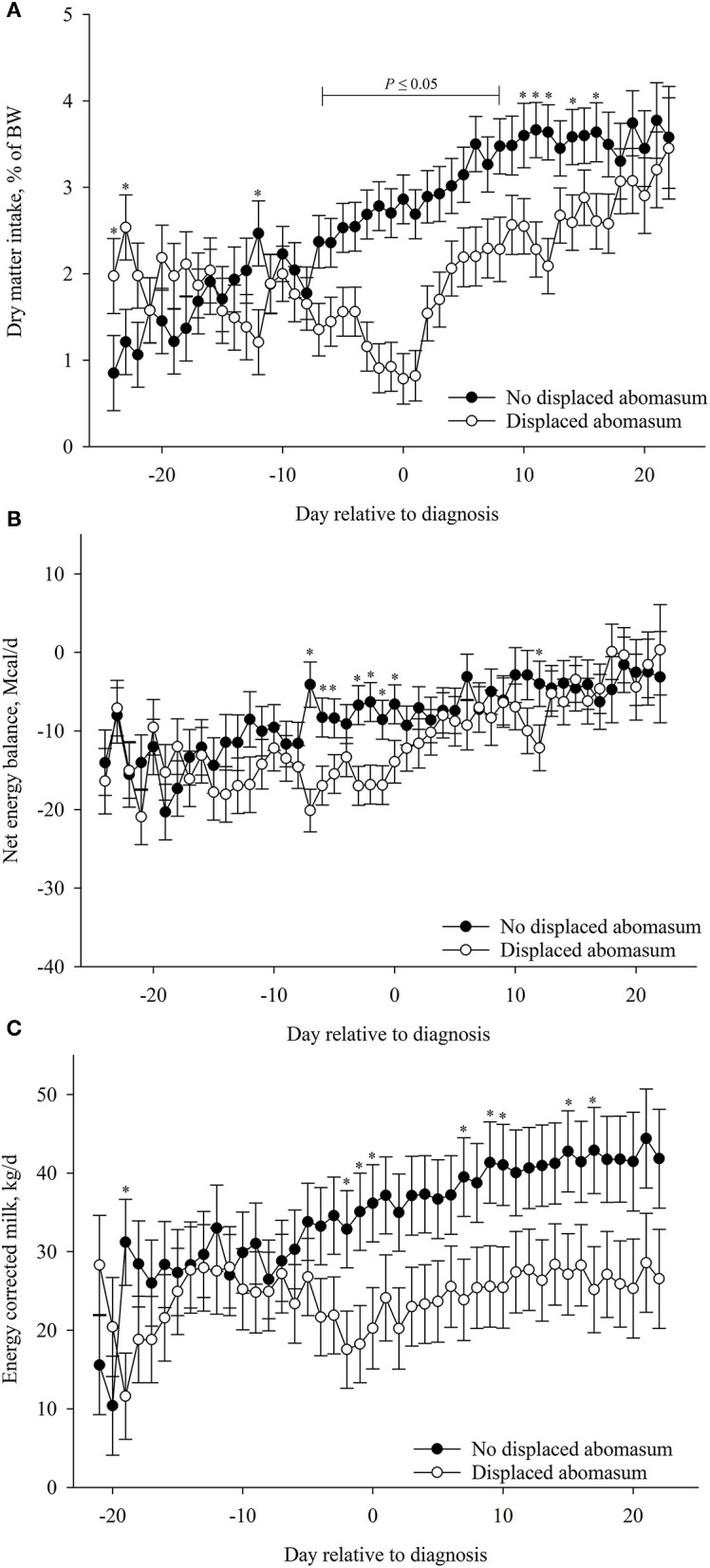
Association of left displaced abomasum postpartum (*n* = 26) with **(A)** dry matter intake (DMI, %BW), **(B)** net energy balance (EB, Mcal/day), and **(C)** energy-corrected milk (ECM, kg/day) during the postpartum period (from −24 to 22 days relative to diagnosis). Values are least square means ± SEM. Postpartum DMI (%BW): left displaced abomasum, *p* = 0.11; day relative to parturition, *p* < 0.01; and the interaction between left displaced abomasum and day, *p* < 0.01. Postpartum EB: left displaced abomasum, *p* = 0.28; day relative to parturition, *p* < 0.01; and the interaction between left displaced abomasum and day, *p* < 0.01. ECM: left displaced abomasum, *p* = 0.17; day relative to parturition, *p* < 0.01; and the interaction between left displaced abomasum and day, *p* < 0.01. *p ≤ 0.05.

The association between LDA and DMI%BW, EB, and ECM were dependent on time (Table 2). Cows that had LDA postpartum had lesser DMI%BW than cows that did not develop LDA on days −24, −23, −12, −7 to 0 and from days 1 to 8 and 10 to 12, 14, and 16 relative to diagnosis (Figure 3A). Cows that had LDA postpartum had lesser EB than cows that did not develop LDA from days −7 to −5, −3 to 0, and 12 relative to diagnosis (Figure 3B). Cows that had LDA postpartum had lesser ECM on days −19, −2, −1, 0, 7, 9, 10, 15, and 17 relative to diagnosis, compared with cows that did not develop LDA (Figure 3C).

### Association of Prepartum DMI%BW and EB With Indigestion

**Figure 4:**
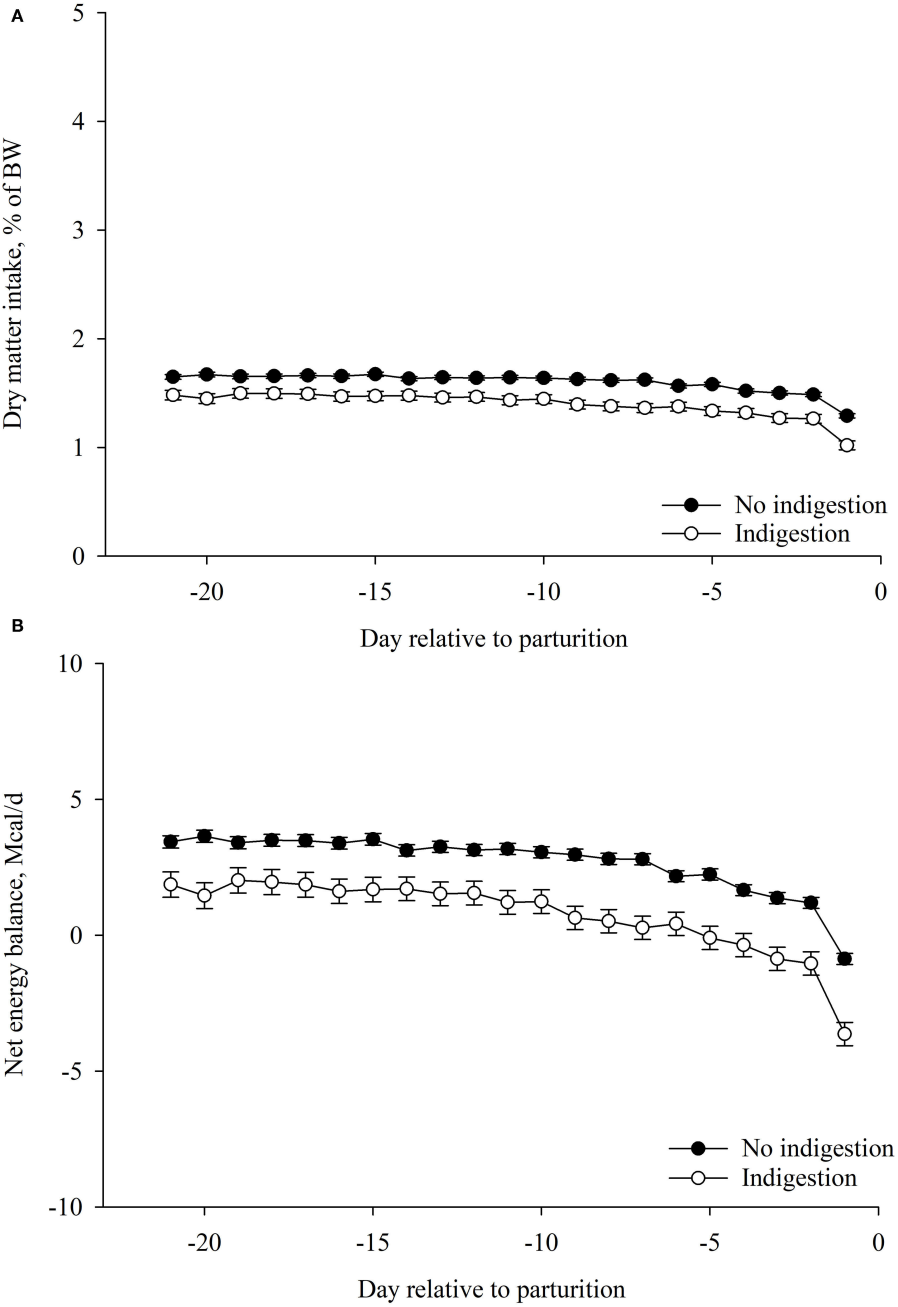
Association of indigestion postpartum (*n* = 118) with **(A)** dry matter intake (DMI, %BW) and **(B)** net energy balance (EB, Mcal/day) during the prepartum period (from −21 to −1 day relative to parturition). Values are least square means ± SEM. Prepartum DMI (%BW): indigestion, *p* < 0.01; day relative to parturition, *p* < 0.01; and the interaction between indigestion and day, *p* = 0.09. Prepartum net energy balance: indigestion, *p* < 0.01; day relative to parturition, *p* < 0.01; and the interaction between indigestion and day, *p* = 0.04. *p ≤ 0.05.

Cows that developed indigestion had lesser prepartum DMI%BW (*p* < 0.01) compared with cows that did not develop indigestion (Table 4; Figure 4A). Cows that developed indigestion had lesser prepartum EB (*p* < 0.01) compared with cows that did not developed indigestion (Table 4; Figure 4B).

**Table 4:** Association of pre- (−21 to −1 days) and postpartum (1 to 28 days) dry matter intake as percentage of body weight (DMI%BW), energy balance (EB), and energy-corrected milk (ECM) with indigestion (Ind) postpartum according to multivariable analysis.

	**Prepartum**		***p*** **-Value**			**Postpartum**		***p*** **-Value**		
	**Ind**	**No Ind**	**Ind**	**Day**	**Ind × day**	**Ind**	**No Ind**	**Ind**	**Day**	**Ind × day**
DMI%BW	1.43 ± 0.03	1.63 ± 0.02	<0.01	<0.01	0.09	2.42 ± 0.07	2.37 ± 0.08	0.58	<0.01	<0.01
EB (Mcal/day)	0.74 ± 0.4	2.7 ± 0.2	<0.01	<0.01	0.04	−7.7 ± 0.7	−8.00 ± 0.8	0.80	<0.01	0.38
ECM (kg/day)	–	–	–	–	–	32.1 ± 1.3	32.8 ± 1.3	0.72	<0.01	<0.01

*Day, day relative to parturition; Ind × day, interaction between indigestion and day*.

### Prepartum DMI%BW and EB as Predictors of Indigestion

Of the variables evaluated, the average of DMI%BW and EB during the last 3 days prepartum, body condition score, and heat stress abatement were predictors of indigestion postpartum.

For each 0.1 percentage point decrease in the average DMI%BW in the last 3 days prepartum, the odds of having indigestion increased by 9% (OR, 1.09; CI, 1.04–1.15), and for each Mcal decrease in the average EB in the last 3 days prepartum, the odds of having indigestion increased by 9% (OR, 1.09; CI, 1.04–1.14; Table 3). The average DMI%BW ranged from 0.27 to 2.90%, with an interquartile range from 1.03 to 1.70%, and the average EB ranged from −16.72 to 25.12 Mcal/day, with an interquartile range from −0.82 to 5.47 Mcal/day.

Cows with high BCS had increased odds of developing indigestion postpartum compared with cows with low BCS (OR, 2.2; CI, 1.5–3.6). Cows in cool weather had increased odds of developing indigestion postpartum compared with cows under heat stress with evaporating cooling (OR, 1.76; CI, 1.1–2.9); whereas, there was no difference in the odds of developing indigestion for cows under heat stress without evaporating cooling compared with cows under heat stress with evaporating cooling (OR, 1.25; CI, 0.61–2.5).

When the average DMI%BW and EB in the last 3 days prepartum were included individually in the indigestion-predicting models containing BCS and heat stress abatement, the AUC increased from 0.60 (CI, 0.56–0.65) to 0.64 (CI, 0.60–0.69) and the AUC were different (*p* < 0.05) between the models.

The average DMI%BW and EB during the last 3 days prepartum produced cutoffs (*p* < 0.01) to predict indigestion, which were ≤1.3 DMI%BW and ≤0.68 EB (Table 5).

**Table 5:** Cut-offs of dry matter intake DMI as percentage of BW (DMI%BW) and energy balance (EB) to predict indigestion postpartum.

	**Cut-off**	**Se (%)**	**Sp (%)**	**PPV (%)**	**NPV (%)**	**Acc (%)**	**AUC**	***p*-Value**
DMI%BW	≤1.3	65	55	23	88	57	0.61	<0.01
EB (Mcal/day)	≤0.68	74	48	23	90	53	0.62	<0.01

*Se, sensitivity; Sp, specificity; PPV, positive predicted value; NPV, negative predictive value; Acc, accuracy; AUC, area under the curve*.

### Association of Postpartum DMI%BW, EB, and ECM With Indigestion

**Figure 5:**
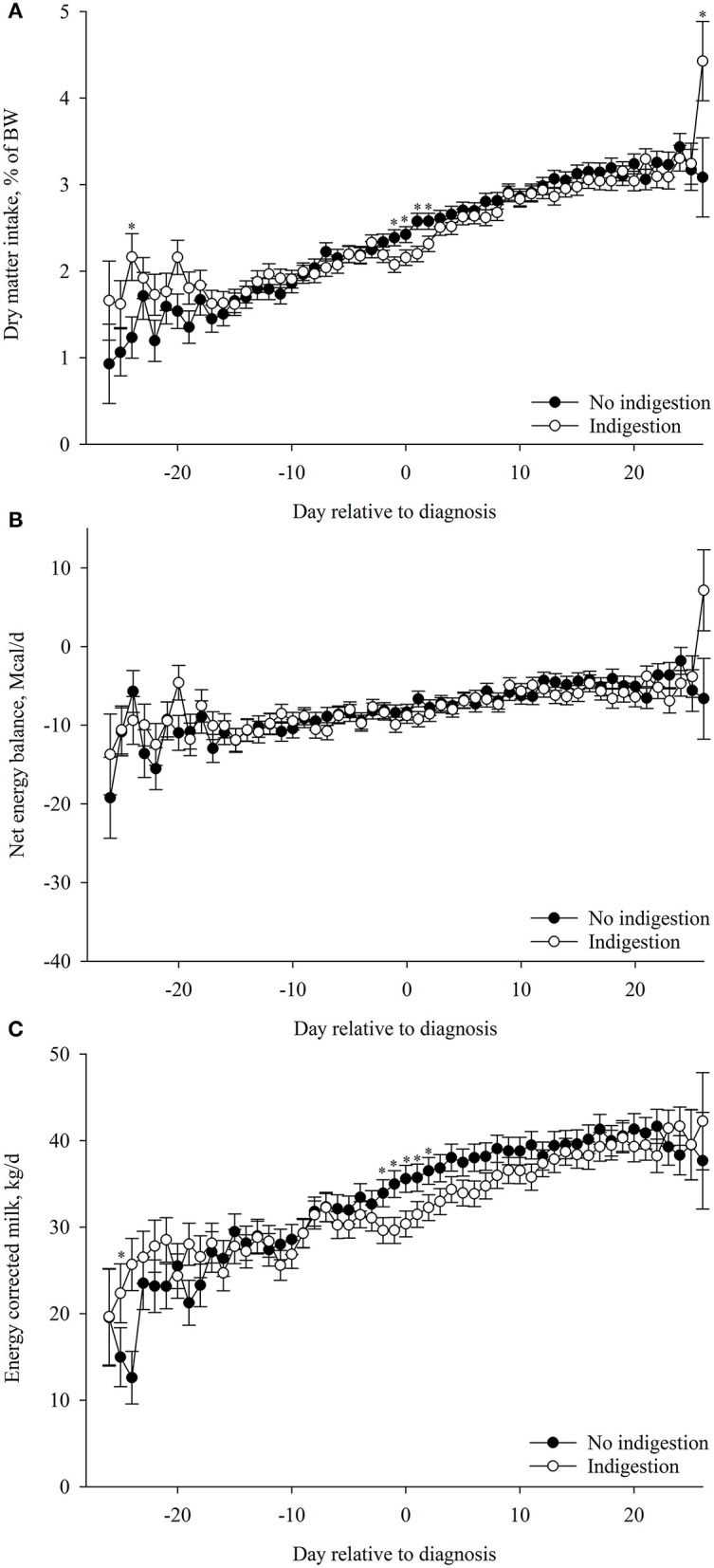
Association of indigestion postpartum (*n* = 118) with **(A)** dry matter intake (DMI, %BW), **(B)** net energy balance (EB, Mcal/day), and **(C)** energy-corrected milk (ECM, kg/day) during the postpartum period (from −26 to 26 days relative to diagnosis). Values are least square means ± SEM. Postpartum DMI (%BW): indigestion, *p* = 0.58; day relative to parturition, *p* < 0.01; and the interaction between indigestion and day, *p* < 0.01. Postpartum EB: indigestion, *p* = 0.80; day relative to parturition, *p* < 0.01; and the interaction between indigestion and day, *p* = 0.38. ECM: indigestion, *p* = 0.72; day relative to parturition, *p* < 0.01; and the interaction between indigestion and day, *p* < 0.01.*p ≤ 0.05.

The association of postpartum DMI%BW with indigestion was dependent of time (*p* < 0.01; Table 4). Cows that had indigestion had lesser postpartum DMI%BW than for cows that did not develop indigestion on days −24, −1, 0, 1, and 2 and greater DMI%BW on day 26 relative to diagnosis (Figure 5A). Postpartum EB was not associated (*p* = 0.80) with indigestion (Table 4; Figure 5B). The association of ECM with indigestion was dependent of time (*p* < 0.01; Table 4). Cows that had indigestion had lesser ECM than cows that did not develop indigestion on days −24, −2, −1, 0, 1, and 2 (Table 4; Figure 5C).

### Association of Prepartum DMI%BW and EB With ODDZ

**Figure 6:**
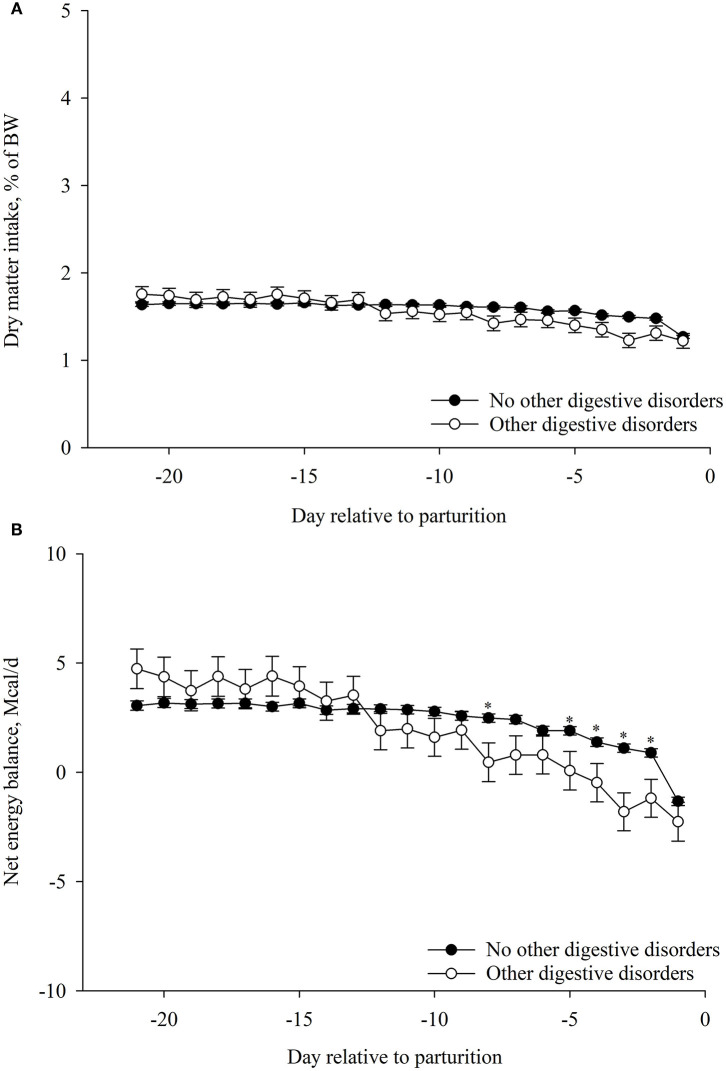
Association of other digestive disorders postpartum (*n* = 31) with **(A)** dry matter intake (DMI, %BW) and **(B)** net energy balance (EB, Mcal/day) during the prepartum period (from −21 to −1 day relative to parturition). Values are least square means ± SEM. Prepartum DMI (%BW): other digestive disorders, *p* = 0.51; day relative to parturition, *p* < 0.01; and the interaction between other digestive disorders and day, *p* < 0.01. Prepartum net energy balance: other digestive disorders, *p* = 0.53; day relative to parturition, *p* < 0.01; and the interaction between other digestive disorders and day, *p* < 0.01. *p ≤ 0.05.

The association of prepartum DMI%BW with ODDZ was dependent of time (*p* < 0.01; Table 6). Cows that had ODDZ had lesser prepartum DMI%BW on day −8 and from days −5 to −2 compared with cows that did not develop other digestion disorders (Figure 6A). The association of prepartum EB with ODDZ was dependent of time (*p* < 0.01; Table 6). Cows that had ODDZ had lesser prepartum EB on day −8 and from days −5 to −2 compared with cows that did not develop other digestion disorders (Figure 6B).

**Table 6:** Association of pre- (−21 to −1 day) and postpartum (1 to 28 days) dry matter intake as percentage of body weight (DMI%BW), energy balance (EB), and energy-corrected milk (ECM) with other digestion disorders (ODDZ) postpartum according to multivariable analysis.

	**Prepartum**		***p*** **-Value**			**Postpartum**		***p*** **-Value**		
	**ODDZ**	**No ODDZ**	**ODDZ**	**Day**	**ODDZ × day**	**ODDZ**	**No ODDZ**	**ODDZ**	**Day**	**ODDZ × day**
DMI%BW	1.55 ± 0.07	1.59 ± 0.02	0.51	<0.01	<0.01	2.23 ± 0.15	2.68 ± 0.15	0.04	<0.01	0.37
EB (Mcal/day)	1.9 ± 0.7	2.4 ± 0.2	0.53	<0.01	<0.01	−7.4 ± 1.7	−5.4 ± 1.7	0.40	<0.01	0.53
ECM (kg/day)	–	–	–	–	–	30.5 ± 1.9	34.3 ± 1.9	0.57	<0.01	0.96

*ODDZ, other digestive disorders that include sand impaction, cecal dilation, bloat, diarrhea, and constipation; Day, day relative to parturition; ODDZ × day, interaction between other digestive disorders and day*.

### Prepartum DMI%BW and EB as Predictors of ODDZ

The average DMI%BW and EB during the last 3 days prepartum were not explanatory variables for ODDZ (Table 3). Of the variables evaluated, parity, BCS, and heat stress abatement were the only predictors of ODDZ postpartum. Multigravid cows had 3.8 times increased odds of developing ODDZ postpartum compared with primigravid cows (OR, 3.8; CI, 1.08–13.5). Cows with high BCS had decreased odds of developing ODDZ postpartum compared with cows with low BCS (OR, 0.36; CI, 0.14–0.93). Cows in cool weather had decreased odds of developing ODDZ postpartum compared with cows under heat stress with evaporating cooling (OR, 0.35; CI, 0.14–0.86); whereas, there was no difference in the odds of developing ODDZ in cows under heat stress without evaporating cooling compared with cows under heat stress with evaporating cooling (OR, 0.97; CI, 0.37–2.5).

### Association of Postpartum DMI%BW, EB, and ECM With ODDZ

**Figure 7:**
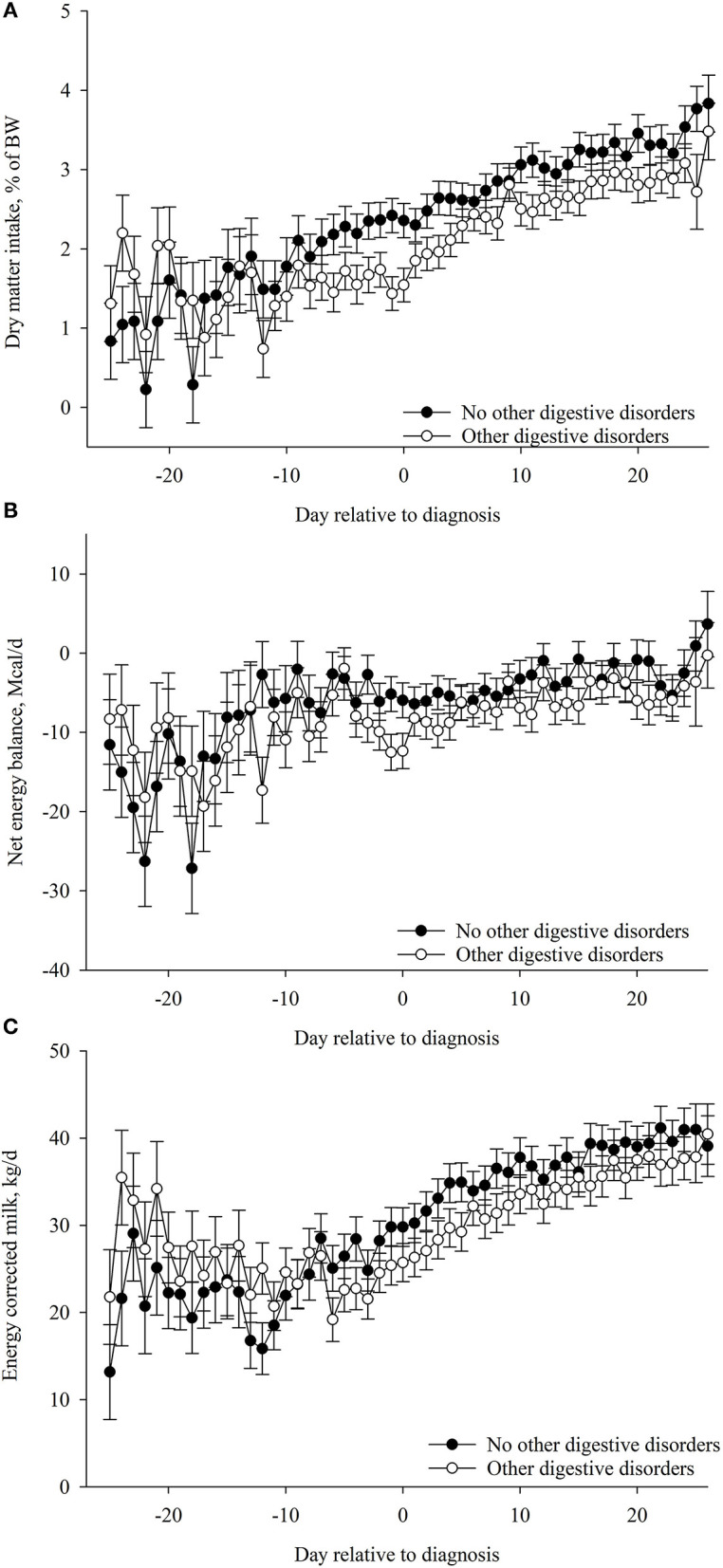
Association of other digestive disorders postpartum (*n* = 31) with **(A)** dry matter intake (DMI, %BW), **(B)** net energy balance (EB, Mcal/day), and **(C)** energy-corrected milk (ECM, kg/day) during the postpartum period (from −25to 25 days relative to diagnosis). Values are least square means ± SEM. Postpartum DMI (%BW): other digestive disorders, *p* = 0.04; day relative to parturition, *p* < 0.01; and the interaction between other digestive disorders and day, *p* = 0.37. Postpartum EB: other digestive disorders, *p* = 0.40; day relative to parturition, *p* < 0.01; and the interaction between other digestive disorders and day, *p* = 0.53. ECM: other digestive disorders, *p* = 0.57; day relative to parturition, *p* < 0.01; and the interaction between other digestive disorders and day, *p* = 0.96.*p ≤ 0.05.

Cows that developed ODDZ had lesser postpartum DMI%BW (*p* = 0.04) compared with cows that did not develop ODDZ (Table 6; Figure 7A). Postpartum EB and ECM were not associated (*p* > 0.40) with ODDZ (Table 6; Figures 7B,C).

## Discussion

In this study, we showed that cows that developed LDA, indigestion, and ODDZ had decreased DMI%BW or EB during the transition period. Furthermore, the average DMI%BW and EB in the last 3 days prepartum were predictive of indigestion, although the effect sizes were small.

As previously stated, there is scant literature on the association of DMI%BW and EB prepartum with digestive disorders postpartum. Janovick et al. (16) showed that cows that were feed restricted prepartum had lesser incidence of LDA postpartum than cows that were fed *ad libitum*. Feed-restricted cows also had decreased lipid mobilization, decreased lipid accumulation in the liver, and decreased ketosis incidence postpartum. Interestingly, feed-restricted cows had decreased circulating concentrations of leptin prepartum, which could have helped maintain DMI pre- and postpartum; therefore, improving metabolism and health. Furthermore, increased NEFA prepartum has been determined to be a risk factor for LDA postpartum (17), which indicated that prepartum DMI%BW and EB could have been negatively impacted in cows that later developed LDA. Herein, we saw that cows that developed LDA had lesser EB prepartum compared with cows that did not develop LDA, and numerically lesser DMI%BW. Nonetheless, neither EB nor DMI%BW could be used to predict LDA postpartum. Therefore, our interpretation is that maintaining DMI and EB prepartum is important for preventing LDA but cannot be used to predict LDA postpartum. This is likely a result of the multifactorial nature of LDA development.

During postpartum, cows that developed LDA had decreased DMI%BW before and on the day of diagnosis, and this decrease continued during the first 2 weeks after diagnosis. Edwards and Tozer (2004) showed that cows with LDA increased activity in the last 10 days prior to diagnosis which could mean less time eating at the feed bunk, therefore, lower DMI%BW before the onset of LDA. Energy balance was also reduced in cows with LDA and indigestion which might be a consequence of lower DMI and the onset of lactation. Furthermore, this decrease in DMI and increase in NEFA can lead to subclinical ketosis which is a risk factor for LDA (17) and consequently exacerbate the decrease in postpartum DMI%BW and EB (3, 48). Energy-corrected milk in cows that developed LDA was lesser from day −2 relative to diagnosis and continued to be decreased up to day 17 after diagnosis. In agreement to our results, Edwards and Tozer (49) showed that milk yield for cows with LDA starts to decline ~3 days before diagnosis and continued to decrease during the first 7 days after diagnosis compared with healthy cows.

Cows with indigestion had decreased prepartum DMI%BW and EB. Furthermore, DMI%BW and EB in the last 3 days prepartum were significant predictors for indigestion postpartum, although the contribution to the prediction was modest. This indicates that DMI%BW and EB prepartum are predictors of indigestion postpartum, but their contribution is minor when accounting for other variables such as BCS and heat stress abatement. A limitation of the current study is that we did not perform external validation of our predictive models; therefore, future validation studies are needed. Herein, we calculated EB prepartum but others have used BCS change prepartum as a proxy for EB and found that cows that had loss of BCS prepartum had greater incidence of indigestion and uterine disease postpartum (18). In addition, we determined cutoffs for DMI%BW and EB to see if they could be used solely as a predictor of indigestion postpartum, and the cutoffs resulted in low to moderate sensitivity, specificity, overall accuracy, and AUC. Therefore, although significant, these cutoffs are of limited applicability. In summary, DMI%BW and EB prepartum are significant but minor contributors to indigestion development postpartum and cannot be used reliably to identify cows that will develop indigestion postpartum.

During postpartum, we showed that cows with indigestion decreased postpartum DMI%BW 1 day before diagnosis, and the decrease continued during the 3 days after diagnosis. (50) showed that there is a decrease in rumination in cows that developed indigestion at least 5 days before clinical diagnosis, indicating that ruminal activity and therefore a decrease in DMI%BW and EB occurred before the onset of clinical diagnosis postpartum. Energy-corrected milk in cows that developed indigestion was lesser from day −2 relative to diagnosis and continued to decrease up to day 2. Similar to our results, Kirchman et al. (9) showed that cows diagnosed with indigestion had decreased milk yield on the day of diagnosis compared with healthy cows. In addition, other studies where indigestion was lumped with other digestive disorders, milk yield was shown to decrease before and after disease diagnosis compared with healthy cows (49, 51).

Cows with ODDZ also had lesser prepartum DMI%BW and EB in the last 5 days prepartum. However, the average of DMI%BW and EB during the last 3 days prepartum were not predictors of ODDZ. In addition, during postpartum, we showed that cows with ODDZ had lesser DMI%BW compared with cows that did not developed ODDZ during postpartum, and most of the differences occurred around the time to diagnosis. Previous research showed that cows that developed digestive disorders, which included indigestion and LDA, had increased activity at 8 days prior to disease diagnosis postpartum compared with healthy cows (49). Hence, if cows that developed digestive disorders spent more time walking, they probably spent less time eating before disease diagnosis. After disease diagnosis, their activity was lesser than healthy cows, which could mean they spend more time laying down and not eating. Unfortunately, they did not evaluate rumination data. The results of this study and previous studies (2, 3) indicate that maintaining DMI%BW during the last days of prepartum could reduce postpartum disorders.

An interesting finding of this study is that ODDZ were associated with reduced ECM. These results are different from what has been reported by others. (52) showed that cows with digestive disorders, excluding diarrhea and DA, had decreased milk production from −4 to 35 days relative to the day of diagnosis compared with cows that did not have the disease event. Edwards and Tozer (49) observed that cows that developed disease postpartum (i.e., at least one of the following: ketosis, RP, milk fever, LDA, indigestion, acidosis, and bloating, reduced feed intake or hardware disease) produced an average of 2.1 kg/day less milk than healthy cows. In this case, the effect of having a digestive disorder cannot be isolated from other diseases or disorders. Indeed, we have observed that cows that had calving disorders, metritis, and clinical mastitis postpartum had decreased milk yield, whereas cows that had ketosis had increased milk yield compared with cows that did not have those diseases or disorders (2, 3). Others have looked at management factors pre- and postpartum that may affect milk production and found that the most important non-dietary factors that affected milk production were age at first calving, presence or absence of feed refusals, number of free stalls per lactating cow, and whether feed was pushed up in the feed bunk (53). These findings show that several factors not accounted for in this study could have affected milk yield.

In conclusion, this study showed that digestive disorders such as indigestion and ODDZ were associated with prepartum DMI%BW and EB whereas LDA was associated with prepartum EB. The average DMI%BW and EB in the last 3 days prepartum were significant explanatory variables for indigestion, and the average DMI%BW and EB in the last 3 days prepartum increased the predictive ability of indigestion although the effect sizes were small. Prepartum cutoffs for DMI%BW and EB to predict indigestion postpartum were established, although with low sensitivity, specificity, and overall accuracy. In addition, LDA, indigestion, and ODDZ were associated with postpartum DMI%BW whereas LDA was associated with EB relative to the day of diagnosis. In summary, DMI%BW and EB prepartum are associated with digestive disorders and are significant but minor contributors to the risk of indigestion postpartum.

## Data Availability Statement

The raw data supporting the conclusions of this article will be made available by the authors, without undue reservation.

## Ethics Statement

The animal study was reviewed and approved by Institutional Animal Care and Use Committee, University of Florida.

## Author Contributions

All authors listed have made a substantial, direct and intellectual contribution to the work, and approved it for publication.

## Conflict of Interest

GG, LG, and NM were employed by the companies Merck Animal Health, Kemin Industries Inc, and Zoetis (United States), respectively. The remaining authors declare that the research was conducted in the absence of any commercial or financial relationships that could be construed as a potential conflict of interest.

## Publisher's Note

All claims expressed in this article are solely those of the authors and do not necessarily represent those of their affiliated organizations, or those of the publisher, the editors and the reviewers. Any product that may be evaluated in this article, or claim that may be made by its manufacturer, is not guaranteed or endorsed by the publisher.

## Footnotes

^1^ https://vetmed-extension.sites.medinfo.ufl.edu/files/2012/01/WEB_VERSSION_-2011-Dairy-Unit-Complete-SOPs-vers-11-07-01-with-Title-page.pdf
