# Supplementary material for: Investigating the Use of Dry Matter Intake and Energy Balance Prepartum as Predictors of Digestive Disorders Postpartum
Source: Front Vet Sci. 2021 Sep 16;8:645252. doi: 10.3389/fvets.2021.645252 (PMC8481776; doi:10.3389/fvets.2021.645252)
Supplement: Supplementary file 1 [file Table_1.DOCX]

Table S1. Chemical composition of experimental diets during pre- and postpartum periods fed to cows used in this study.

|  | Prepartum | | | |  | Postpartum | | | |
| --- | --- | --- | --- | --- | --- | --- | --- | --- | --- |
| Experiment | ^1^NE, Mcl/kg of DM | ^2^CP, % of DM | ^3^ADF, % of DM | ^4^NDF, % of DM |  | NE, Mcl/kg of DM | CP, % of DM | ADF, % of DM | NDF, % of DM |
| do Amaral et al. (2009) | 1.52 | 14.6 | 24.9 | 40.2 |  | 1.63 | 17.1 | 20.9 | 32.9 |
| do Amaral et al. (2011) | 1.49 | 12.9 | 24.5 | 41.8 |  | 1.61 | 17.5 | 19.4 | 31.3 |
| Tao et al. (2011) | ^5^NA | NA | NA | NA |  | NA | NA | NA | NA |
| Tao et al. (2012) | NA | NA | NA | NA |  | NA | NA | NA | NA |
| Gomes (2014) | 1.57 | 14.1 | 30 | 44.1 |  | 1.65 | 16.3 | 21.3 | 31.6 |
| Greco (2014) |  |  |  |  |  |  |  |  |  |
| Treatment 1 | 1.42 | 14.0 | 25.6 | 47 |  | 1.59 | 16.7 | 16.8 | 29.5 |
| Treatment 2 | 1.49 | 13.9 | 25.3 | 48.2 |  | 1.67 | 16.3 | 16.2 | 29.1 |
| Treatment 3 | 1.5 | 14.1 | 25.5 | 47.4 |  | 1.67 | 16.4 | 16.8 | 29.9 |
| Thompson et al. (2014) | NA | NA | NA | NA |  | NA | NA | NA | NA |
| Martinez et al. (2018) |  |  |  |  |  |  |  |  |  |
| Treatment 1 | 1.65 | 13.5 | 24.5 | 37.8 |  | 1.67 | 15.7 | 21.5 | 33.3 |
| Treatment 2 | 1.65 | 12.9 | 23.8 | 39 |  | 1.67 | 15.7 | 21.5 | 33.3 |
| Treatment 3 | 1.65 | 13.5 | 23.9 | 38.3 |  | 1.67 | 15.7 | 21.5 | 33.3 |
| Treatment 4 | 1.65 | 13.4 | 24.0 | 38.2 |  | 1.67 | 15.7 | 21.5 | 33.3 |
| Zenobi et al. (2018) |  |  |  |  |  |  |  |  |  |
| Treatment 1 | 1.63 | 14.0 | 26.95 | 43.2 |  | 1.68 | 16 | 24 | 37.1 |
| Treatment 2 | 1.4 | 13.5 | 36.2 | 55.7 |  | 1.68 | 16 | 24 | 37.1 |
| Lopera et al. (2018) |  |  |  |  |  |  |  |  |  |
| Treatment 1 | 1.46 | 14.9 | 29.4 | 43.1 |  | 1.66 | 17.4 | 22.5 | 31.6 |
| Treatment 2 | 1.45 | 14.7 | 28.9 | 43.7 |  | 1.66 | 17.4 | 22.5 | 31.6 |
| Treatment 3 | 1.45 | 14.6 | 29.1 | 43.8 |  | 1.66 | 17.4 | 22.5 | 31.6 |
| Bollati et al. (2020) | 1.54 | 15.8 | 25.9 | 45.4 |  | 1.66 | 16.6 | 19.9 | 29.9 |

^1^NE = Net energy; ^2^CP = Crude protein; ^3^ADF = Acid detergent fiber; ^4^NDF = Neutral detergent fiber; ^5^NA = Not available.
